# Early Childhood Education and Care Enhances Cognitive Performance in Later Adolescence Through Non-Cognitive Skills Development and Reduced Truancy

**DOI:** 10.3390/jintelligence13120164

**Published:** 2025-12-15

**Authors:** Ji Liu, Millicent Aziku, Dahman Tahri

**Affiliations:** Faculty of Education, Shaanxi Normal University, Xi’an 710062, China; millyzi78@snnu.edu.cn (M.A.); dahmantahri@snnu.edu.cn (D.T.)

**Keywords:** early childhood education and care, non-cognitive skills, truancy, cognitive performance, mediation analysis

## Abstract

Prior studies have examined associations between early childhood education and care (ECEC) and cognitive performance in later adolescence. However, little is known about the role of non-cognitive skills development and truancy in this link. To address this gap, the current study investigates how non-cognitive skills and truancy mediate the link between ECEC and cognitive performance among 15-year-old students (N = 550,818), leveraging the Programme for International Student Assessment (PISA) 2022 dataset. Findings indicate that ECEC directly and positively influences non-cognitive skills development and cognitive performance. Non-cognitive skills development is negatively associated with truancy and positively influences cognitive performance. An inverse relationship was found between truancy and cognitive performance. Analyzing this relationship based on gender, it was observed that female students benefited more from ECEC compared to their male counterparts. These results imply that the provision of ECEC may reap substantial social equity benefits.

## 1. Introduction

Scholars, educators, and policymakers have long been interested in the cognitive performance of students across all levels of education (Xie et al. 2023). Cognitive performance usually refers to the mental process and capability of exhibiting acquired knowledge through problem-solving, reasoning, and comprehension (Seblova et al. 2020). Often, academic success determines future educational attainment and broader life outcomes. High-level cognitive performance is a critical indicator for better job opportunities and participation in decision-making, consequently resulting in better socioeconomic status for not only for individuals but also their families (Lurie et al. 2021).

Extant studies have emphasized consistent promotion of early childhood education and care (ECEC) for all students, arguing that the number of years an individual spent in ECEC is directly linked with the person’s cognitive performance; therefore, it is important for all learners to start their formal education at an early stage (Lövdén et al. 2020; Thümmler et al. 2022). Studies explained that students who attended ECEC programs developed mechanisms and cues for overcoming academic anxiety (Dunkel and Murphy 2014). Betancur et al. (2024) argued that ECEC was a contributing factor to bridging existing cognitive achievement gap between rural and urban learners, in that if rural students enrolled in ECEC programs as their urban peers did, their cognitive performance in adolescence would improve as well.

Relatedly, prior studies underscore the key role of non-cognitive skills such as assertiveness, perseverance, cooperation, curiosity, emotional control, and stress resistance in promoting cognitive performance. For instance, a study by Evans et al. (2023) described curiosity as an important driver for cognitive skills development and suggested that it needs to be promoted among learners of all ages through social interactions and student engagement. Stress resistance, emotional regulation, and perseverance are recognized as essential facilitators for establishing conducive learning environments that help students get past their learning obstacles and persevere through academic difficulties (Sannathimmappa et al. 2022; Tan et al. 2023; Xu et al. 2023). 

Relatedly, regular truancy is detrimental for cognitive performance (Çali et al. 2024; Maynard et al. 2012). Truancy is a prevalent issue that not only puts students at risk of dropping out of school but also negatively impacts their overall school engagement (Lázaro et al. 2020). As students repeatedly absent themselves from school, they disengage from class assignments, homework, and revision exercises (Ampofo et al. 2022). Consequently, counseling against truancy is broadly suggested as a viable technique for improving students’ academic engagement, leading to empowered cognitive performance (Sulaiman and Uhuegbu 2021). A recent study proposed establishment of feasible policy measures that target punctuality and leaner engagement as key interventions that would improve cognitive performance (Çali et al. 2024).

Although previous research has established links between early childhood education and later academic achievement, much less is known about the mechanisms underlying this relationship, particularly the role of non-cognitive skills and truancy. The current study makes three distinct contributions. First, it leverages the most recent Program for International Student Assessment (PISA) 2022 dataset with over 550,818 students across 65 countries, providing robust evidence on the long-term significance of ECEC. Second, it goes beyond direct associations by modeling the mediating roles of non-cognitive skills and truancy, thus revealing indirect pathways that explain how ECEC influences later academic outcomes. Third, it offers a gender-disaggregated analysis, showing whether boys and girls benefit differently from early educational experiences. 

## 2. Literature Review

### 2.1. Relationship Between ECEC and Cognitive Performance

Several recent studies have established a positive relationship between participation in early childhood education and cognitive performance in later stages of education. For instance, Tan et al. (2023) conducted research with 506 kids enrolled in 17 private ECEC centers and suggested that it is imperative to foster fundamental cognitive skills at early stages of education. Another study also found a positive correlation between ECEC and cognitive development at later stages of education (Koshy et al. 2024). This explains how important ECEC is in preparing students for future academic success and, in turn, future opportunities. For example, a study that investigated the cognitive performance of students from diverse socioeconomic backgrounds explained that participation in ECEC programs increased the chances of improving students’ performance, hence, bridging the gap (Betancur et al. 2024). In addition, they concluded that ECEC participation could be utilized by countries as a key policy lever for closing rural-urban achievement gap. This assertion is congruent with the claim that the number of years of formal education completed by individuals is positively correlated with their cognitive function throughout adulthood (Feldberg et al. 2024; Lövdén et al. 2020). While existing literature provides evidence on the positive relationship between ECEC and cognitive performance, there still exists a gap that calls for attention. Particularly, little is known about the role that non-cognitive skills and behavioral factors, such as truancy, play in this relationship. 

### 2.2. Relationship Between ECEC and Non-Cognitive Skills

Enrolling students in ECEC programs is regarded as an important measure for building their non-cognitive skills, such as perseverance, curiosity, emotional control, assertiveness, stress resistance, and cooperation in their later academic journeys (Korsvold and Nygård 2022; Thümmler et al. 2022). A longitudinal study by Williams et al. (2022) found that students who had ECEC were more resilient academically. Generally, students develop internal protective factors such as perseverance, assertiveness, and emotional control as a result of early educational experiences, which are prerequisites for academic resilience (Saitadze 2021). Such development is possible because, through ECEC programs, teachers impart valuable foundational non-cognitive regulation skills through structured play, social interaction, and guided learning routines. The routine practices help to build in children habits such as appropriate expression of emotions, disagreement, and anger, and accepting habits such as endurance and persistence (Morando and Platt 2022; Ward et al. 2022). 

### 2.3. Relationship Between ECEC and Truancy

Students who experience formal ECEC develop educational engagement and competencies that enable them to cope with factors likely contributing to adolescent truancy (Wei et al. 2023). Studies show that ECEC teachers actively model the behavior of students and instill in them positive attitudes towards school, thereby encouraging punctuality and regularity (Juaristi et al. 2024; Riggleman et al. 2025). Sangsuk and Thipchart (2023) found a significant positive relationship between ECEC and behavior transformation, such as consistency in schooling engagement. Behavior transformation is ensured because of discipline and motivational measures practiced by teachers and caregivers. Once this is instilled in the students at a tender age, it becomes a habit even until adolescence because they develop routine time management and regular school attendance in later years. Another study s indicated that young children uphold explicit time norms in ECEC programs because of the expectations and subtle norms set by their teachers, which serve as tools for behavioral regulations and transitions from spontaneity to school concentration mode (Schmidt and Li 2025). Furthermore, a study showed that through ECEC exposure, children develop a sense of belonging and see school as their second home, where they bond with their classmates, hence decreasing incidences of truancy (Zhou et al. 2023). Quality relationships that teachers have with students assures the students of their security in the school environment and increases their inclination to punctuality (Saitadze 2021).

### 2.4. Relationship Between Non-Cognitive Skills and Cognitive Performance 

Non-cognitive skills can be described as certain intangible or relational skills that students develop in order to live in coherence with others (Quieng et al. 2015; Zhou 2017; Liu et al. 2024). Research showed a positive relationship between non-cognitive skills and cognitive performance (Pedersen 2020). For example, a research that utilized a large-scale international dataset to explore how non-cognitive skills affected students’ cognitive achievement concluded that the more students report developing non-cognitive skills such as perseverance, projective judgment, and curiosity, the higher their cognitive scores (Lee and Stankov 2018). He et al. (2021) postulated that non-cognitive skills were linked with cognitive performance, and this relationship was more pronounced for average-performing students than for students with lower cognitive abilities generally. In a study conducted in Italy with some 8th-grade students to investigate the factors that influence their cognitive performance and school efficiency, it was discovered that students’ non-cognitive skills largely influenced their performance on cognitive tasks (Vittadini et al. 2022). Furthermore, a study that evaluated the role of non-cognitive skills in the development of cognitive performance of Kazakhstan students indicated that non-cognitive skills either had direct or indirect impacts on students’ cognitive performance, hence, academic achievement (Sultanova et al. 2024). According to Evans et al. (2023), non-cognitive skills are important drivers for cognitive skills development; therefore, they should be promoted among learners of all ages through social interactions and student engagement. In sum, to enhance students’ cognitive performance, there is a need to reinforce their non-cognitive skills to enable them to leverage such skills when they face academic difficulties.

### 2.5. Relationship Between Truancy and Cognitive Performance

Behavioral researchers defined truancy as a consistent skipping of school without valid reason or permission (Maynard et al. 2012; Shute and Cooper 2015; Weathers et al. 2021). Reid (2017) described truancy as a social problem that is riddled with multiple challenges that affect educational outcomes. Truancy was constantly found to have an adverse effect on cognitive performance. For example, a study found that some students performed very low on cognitive tasks as a result of prevalent truancy (Lázaro et al. 2020). As students skip school and classes, a curricular gap is created in their learning experiences, making it difficult for them to excel academically. Therefore, it is essential to employ measures that would aid early detection of the behavior and possible action to deter it. Another study conducted by Ampofo et al. (2022) with some 206 junior high school students in Ghana to determine the cause of poor cognitive performance revealed truancy as a primary issue which did not only put students at the risk of dropping out from school but also predicted their performance as they disengaged from class assignment, HomeWorks and revision exercises. Similarly, a study demonstrated that truancy was a root cause of repeating grades, as students did not attain expected cognitive outcome level that would enable them to be promoted to a higher grade on the academic ladder (Sulaiman and Uhuegbu 2021). This is troubling because constantly repeating a grade leads to reduced academic interest and may also result in school dropout (Boccio et al. 2025). Result from an empirical study suggested that making policies that target punctuality and learner engagement is a key intervention that would improve cognitive performance at the end (Çali et al. 2024).

### 2.6. Potential Indirect Pathways

Extant studies showed that, although some students participated in ECEC programs at a young age, they do not exhibit enhanced cognitive performance (Feldberg et al. 2024; He et al. 2021; Lau et al. 2025). The literature review identifies that non-cognitive skills can be utilized as a key tool for strengthening the relationship between ECEC and cognitive performance (Evans et al. 2023). Enhancing non-cognitive skills in students is likely to reduce the academic struggles students face, hence resulting in intrinsic motivation for continual improvement. This implies that, if these skills are not well nurtured, they may interfere with the relationship between ECEC and cognitive performance (Boccio et al. 2025; Riggleman et al. 2025). This explains the importance of integrating non-cognitive skills development into ECEC curricula and maintaining support throughout students’ academic progression to maximize cognitive benefits of early educational interventions.

Relatedly, despite the inverse correlation that exists between truancy and cognitive performance, educators can enhance students’ academic outcomes through the promotion of ECEC participation before primary education (Bustamante et al. 2023; Lázaro et al. 2020). When truancy is reduced, it results in promoting sustained academic engagement, enabling students to perform higher on cognitive tasks. This discourse demonstrates an influential chain whereby attending ECEC programs refines non-cognitive skills, which in turn discourages truancy, hence augmenting cognitive performance. Specifically, non-cognitive skills and truancy may explain the link between ECEC and cognitive performance.

### 2.7. The Current Study

**Figure 1:**
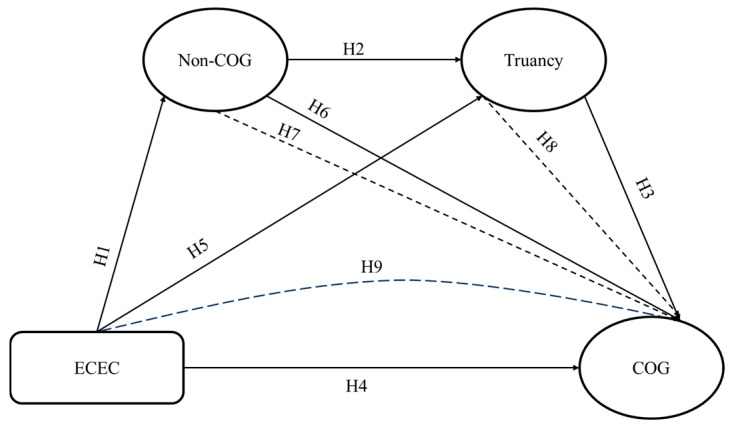
Conceptual diagram of relationships.

With insights drawn from a systematic literature review, the current study investigates the relationship between ECEC and cognitive performance among students who took part in PISA 2022. The study further explores possible mediating roles of non-cognitive skills and truancy in the relationship between ECEC and cognitive performance. This study postulates that early learning environments build the foundation for later cognitive and non-cognitive development, while children’s behaviors serve as key mechanisms linking early experiences to later achievement (Bronfenbrenner 1999; Heckman 2006). Based on the above theoretical perspectives, we hypothesize the following and formalize a conceptual model in guiding the analyses (see Figure 1).

**Hypothesis** **1.** *There is a direct positive and significant relationship between ECEC and non-cognitive skills*.

**Hypothesis** **2.** *There is a direct negative relationship between non-cognitive skills and truancy*.

**Hypothesis** **3.** *Truancy has a negative and direct effect on cognitive performance*.

**Hypothesis** **4.** *There is a direct positive and significant relationship between ECEC and cognitive performance*.

**Hypothesis** **5.** *ECEC has a direct negative and significant relationship with truancy*.

**Hypothesis** **6.** *Non-cognitive skills is positively and directly linked with cognitive performance*.

**Hypothesis** **7.** *ECEC has an indirect positive influence on cognitive performance through non-cognitive skills*.

**Hypothesis** **8.** *ECEC has an indirect positive effect on cognitive performance through truancy*.

**Hypothesis** **9.** *There is a chain relationship among ECEC, non-cognitive skills, truancy, and cognitive performance*.

## 3. Methodology

### 3.1. Participants

**Figure 2:**
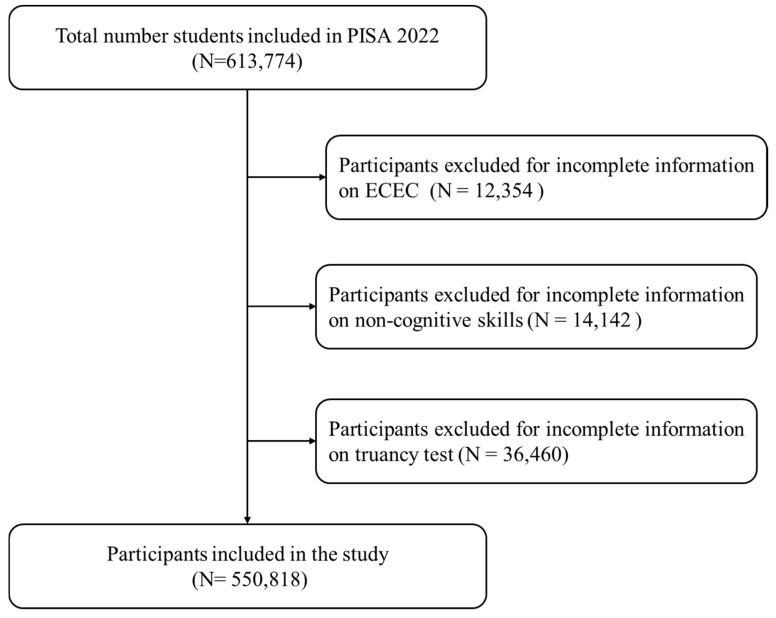
Flow chart of participants inclusion.

This current study utilizes data from the Program for International Student Assessment (PISA) administered by the Organization for Economic Cooperation and Development (OECD) in 2022. PISA is an international, large-scale assessment that surveys 15-year-old students who are enrolled in formal educational institutions at the time of the data collection (OECD 2022). Parental questionnaires were administered to parents or guardians, asking them various questions related to children’s learning conditions, including ECEC. Before answering survey questions, students sat for cognitive tests in science, mathematics, and reading. The PISA dataset is well-suited for the current study because of its extensive nature, which enables the researchers to conduct international analysis of how ECEC impacts adolescent cognitive performance. The current study’s initial sample consisted of 613,744 students from 65 participating countries and economies. However, we excluded participants for either incomplete data on ECEC, non-cognitive performance, or truancy modules. No missing result was observed for background information and cognitive performance. After the exclusion, 550,818 students met the criteria for inclusion in the current study. The exclusion process is presented in Figure 2 below. Table 1 presents the demographic characteristics of participants included in the study.

### 3.2. Ethical Considerations

This study used secondary, open-access data provided by the OECD through PISA. All data are fully anonymized, and no identifiable information about individual students or schools is included. As a result, no ethical committee approval or additional informed consent was required for this research.

### 3.3. Instruments

#### 3.3.1. Early Childhood Education and Care

The key independent variable in this study is early childhood education and care, which is refers to as all arrangements providing care and/or educational activities for young children before they enter primary school. ECEC was measured using a construct adapted from a three-item scale in the PISA 2022 parent questionnaire. Parents were asked whether their children regularly attended one of the following before grade 1: (1) Supervision and care, which refers to non-formal childcare services whose primary purpose is to look after young children rather than to deliver a structured curriculum (e.g., <national examples>), (2) Early childhood educational development (e.g., <national examples>), and (3) Pre-primary education (e.g., <national examples>).

To estimate the intensity of ECEC participation, the current study combined the reported duration and weekly participation time, adjusting for country-specific differences in primary-school entry. Specifically, the statutory legal starting age of primary school in each country was taken from OECD data on educational access (OECD 2023), and merged with PISA dataset. Total number of ECEC hours received by student *i* in country *c* is estimated as a function of the following:

$${\mathrm{ECEC}}_{ic\;}=\left({SA}_{c}-{\mathrm{SA}}_{i}\right)\;\times \;{\mathrm{WH}}_{i\;}\times \;30$$

where

${SA}_{c}$ = the statutory legal starting age of primary school in country c;

${\mathrm{SA}}_{i}$ = the reported starting age of ECEC for child *i*;

${\mathrm{WH}}_{i}$ = the average number of hours per week in ECEC for child *i*;

30 = the number of weeks in a typical academic year.

#### 3.3.2. Non-Cognitive Skills

The current study conceptualizes non-cognitive skills as a vector of internal psychosocial attributes, including perseverance, stress resistance, emotional control, assertiveness, curiosity, and cooperation, that serve as influential factors for positive academic behavior. In PISA 2022, students rate their agreement with statements about a range of non-cognitive skills development on a five-point Likert scale ranging from “strongly agree” to “strongly disagree” (e.g., “I keep my emotions under control,” “I get mad easily,” “I take initiative when working with my classmates,” “I work well with other people”). “I like to know how things work.” “I keep working on a task until it is finished,” etc. Reverse scoring was applied systematically across all negatively worded items in the non-cognitive skills construct to maintain consistency in interpretation and ensure accurate representation of the underlying traits. For example, “I avoid working together with other students” and “I tend to be selfish” were originally coded so that agreement indicated lower cooperation. These items were reversed before computing scale scores, so that higher values indicate greater cooperation. We report construct validity indices and standardized factor loadings of non-cognitive skills where Cronbach’s ∝ records 0.703, and the Kaiser-Meyer-Olkin (KMO) sampling adequacy test result records 0.824, both demonstrating a good construct validity. The result for standardized factor loadings ranges from 0.556 to 0.862, which is also an indication that the latent construct is empirically valid. Table 2 below shows the mean, standard deviation, and factor loadings of the construct.

#### 3.3.3. Truancy

Truancy is conceptualized as a latent construct and defined as repetitive absenteeism from school. PISA 2022 measures students’ school attendance with three items indicating how often students indulged in absenteeism. For example, (1) ‘I <skipped> a whole school day,’ (2) ‘I <skipped> some classes,’ and (3) ‘I arrived late for school. The items were rated on a four-point Likert scale ranging from “never” to “five or more times.” We report construct validity indices and standardized factor loadings of truancy where Cronbach’s ∝ records 0.841, and the KMO sampling adequacy test result records 0.823, both demonstrating good construct validity. The result for standardized factor loadings ranges from 0.763 to 0.914, which is also an indication that the latent construct is empirically valid. Table 3 below shows the mean, standard deviation, and factor loadings of the construct.

#### 3.3.4. Cognitive Performance

Cognitive performance is the dependent variable in this study and is measured using students’ achievement scores in science, reading, and mathematics as assessed by PISA. Rather than reporting a single test score, PISA provides a set of plausible values (PVs), typically ten per subject area, for each student. These plausible values are drawn from a distribution of possible proficiencies students might exhibit, based on their responses and background characteristics. They are used to provide unbiased population estimates of student achievement in large-scale assessments. We further report construct validity indices and standardized factor loadings of cognitive performance where Cronbach’s ∝ records 0.965, and the KMO sampling adequacy test result records 0.869, both demonstrating good construct validity. Standardized factor loading results ranges from 0.920 to 0.962, which suggest that this construct is empirically valid. Table 4 presents the mean and standard deviation, and factor loadings of cognitive performance.

#### 3.3.5. Covariates

In addition to the primary variables of interest, the current study controlled for a set of background variables that were commonly associated with students outcomes. These include country, age, immigration status, and socioeconomic status (SES). Controlling for these background variables is essential to isolate the unique effects of ECEC, non-cognitive skills, and truancy on cognitive performance.

### 3.4. Data Analysis

The statistical analyses were conducted using STATA version 18.0 (StataCorp LLC, College Station, TX, USA). To achieve the study’s objectives, we employed a partial least squares structural equation model (PLS-SEM) to examine the relationships among ECEC, non-cognitive skills, truancy, and cognitive performance for 550,818 students, and to estimate the mediating effects of non-cognitive skills and truancy. PLS-SEM was chosen because it is particularly well-suited for large samples, complex mediation models, and research designs prioritizing explanatory power and predictive accuracy (Liu et al. 2025b). This approach reduces potential error terms and provides a robust estimation. Model fit was assessed with standard indices, and mediation effects were tested using Delta, Sobel, and Monte Carlo procedures with 5000 bootstraps. To address potential multicollinearity concerns, all variables were mean-centered and standardized (Imai et al. 2010; Liu et al. 2025a). All 10 plausible values for each domain were used to estimate cognitive performance, and analyses were conducted according to OECD (2022) guidelines to account for measurement uncertainty. All analyses were weighted with the final student weight to reflect the complex two-stage stratified sampling design.

In addition, the analysis is conducted separately for male and female students, as prior research suggests that boys and girls may benefit differently from ECEC (Lau et al. 2025; Maynard et al. 2012). Examining gender differences therefore serves as a theoretically informed exploratory extension, providing a more nuanced understanding of the pathways through which ECEC affects later adolescence outcomes.

## 4. Results

### 4.1. Assessment of Measurement Model

The goodness-of-fit statistics of the structural equation model were assessed using the Comparative Fit Index (CFI), Tucker–Lewis Index (TLI), Root Mean Square Error Approximation (RMSEA), Standardized Root Mean Square Residual (SRMR) and Coefficient of Determination (CD). Specifically, the model fit measures are CFI = 0.961, TLI = 0.893, RMSEA = 0.032 and SRMR = 0.038 and CD = 0.846. In near-universal terms, for CFI and TLI to record goodness-of-fit, they are bounded at 0.9 (Hu and Bentler 1999). The commonly accepted threshold for RMSEA is ≤ 0.06, indicating a good model fit, while for SRMR, a value of ≤ 0.08 is generally considered acceptable. For CD, higher values between 0 and 1 indicate a good fit. We therefore conclude that our model is statistically fit for further analysis.

#### 4.1.1. Direct and Indirect Effects

**Figure 3:**
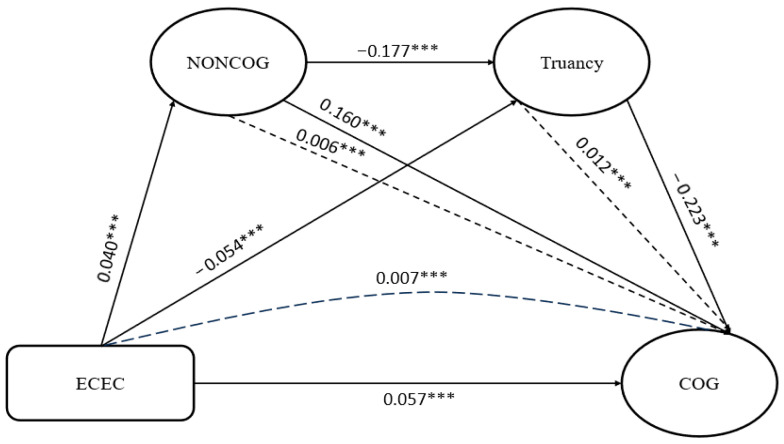
Results of PLS-SEM (Full sample). Note: CFI = 0.961, TLI = 0.893, RMSEA = 0.032 and SRMR = 0.038 and CD = 0.846. *** indicates *p* < 0.001.

The current study explored six pairs of direct pathways and presented the full sample result as a standardized path coefficient (std. β) as shown in Figure 3 and Table 5. The analysis indicates that ECEC is linked to an improvement in non-cognitive skills (std. β = 0.040, *p* < 0.001) and cognitive performance (std. β = 0.057, *p* < 0.001). ECEC also has a direct negative effect on truancy (std. β = −0.054, *p* < 0.001). The result shows that students’ participation in ECEC programs leads to enhancement in both non-cognitive and cognitive skills and reduction in truancy. Assessing the relationship between non-cognitive skills and truancy, the study shows that higher levels of non-cognitive skills are negatively correlated with truancy (std. β = −0.177, *p* < 0.001). The path between non-cognitive skills and cognitive performance is significantly positive (std. β = 0.160, *p* < 0.001), demonstrating that higher non-cognitive skills development correlates with higher cognitive performance. As expected, truancy has a significant negative influence on cognitive performance (std. β = −0.223, *p* < 0.001). The influence of truancy on cognitive performance is larger than that of other relationships, which suggests that students’ attitudes toward school attendance play a substantial role in their academic success. All direct-effect results align with Hypotheses 1–6, and statistically support them

When the indirect pathways were analyzed, results show that non-cognitive skills and truancy significantly mediate the relationship between ECEC and cognitive performance (std. β = 0.006, and 0.012, respectively, *p* < 0.001). That is, the mediated effect via non-cognitive skills is about 10% the direct effect, while the mediated effect via truancy is about 20% the direct effect of ECEC on cognitive performance. This implies that ECEC improves students’ cognitive performance both directly and indirectly by enhancing non-cognitive skills and reducing truancy. The chained indirect effect, which is the effect of ECEC through non-cognitive skills, and then through truancy on cognitive performance, is positive (std. β = 0.007, *p* < 0.001). Meaning that ECEC fosters non-cognitive skills, which in turn lowers truancy and boosts cognitive outcomes. The indirect-effect results provide statistical support for Hypotheses 7–9. This highlights the value of ECEC not only for its direct contribution to learning but also for its role in fostering perseverance and reducing absenteeism, both of which shape long-term academic trajectories.

#### 4.1.2. Gender Differences

**Figure 4:**
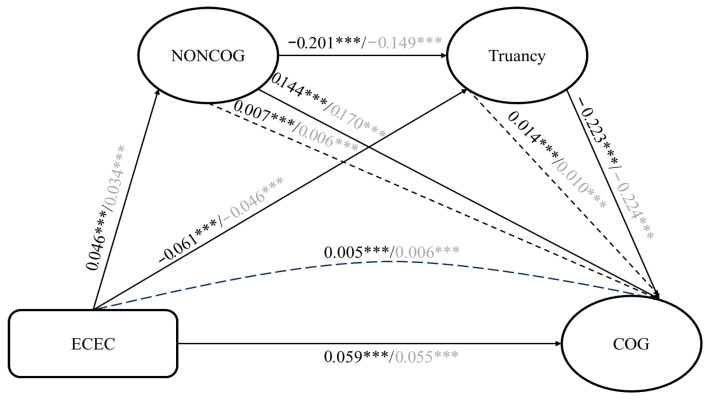
Results of PLS-SEM (Gender). Note: Black font for females and gray font for males. *** indicates *p* < 0.001.

To investigate the possible gender differences, the current study further conducted gender-specific analyses by disaggregating the sample based on gender. As shown in Table 6 and Figure 4, findings highlight consistent gender differences across all examined pathways. For instance, the effect of ECEC on truancy is more pronounced for female students (std. β = −0.061) compared to their male counterparts (std. β = −0.046), suggesting that early education plays a stronger role in reducing truancy among girls. A similar trend is observed in the relationship between ECEC and non-cognitive skills, where the effect size is larger for females (std. β = 0.046) than for males (std. β = 0.034), indicating that girls may be more responsive to early educational interventions in developing socio-emotional and behavioral competencies.

Interestingly, the direct effect of ECEC on cognitive performance also appears slightly stronger for girls (std. β = 0.059) than for boys (std. β = 0.055), reinforcing the idea that early childhood education may yield greater academic returns for female students. Moreover, non-cognitive skills significantly predict truancy for both genders, though the association is stronger for girls (std. β = −0.201) compared to boys (std. β = −0.149), implying that improvements in non-cognitive traits are more likely to reduce absenteeism among female students. The negative impact of truancy on cognitive performance is nearly identical for both genders (std. β = −0.223 for females; −0.224 for males), highlighting the universal detrimental effect of truancy on learning regardless of gender. Non-cognitive skills also exert a direct positive influence on cognitive performance, with a slightly stronger effect among boys (std. β = 0.170) than girls (std. β = 0.144), suggesting that while girls may benefit more behaviorally, boys may leverage non-cognitive development more strongly for academic performance.

The indirect effects further affirm the gendered pathways. For instance, the indirect path ECEC → Truancy → Cognitive Performance is stronger for girls (β = 0.014) than for boys (β = 0.010), and the ECEC → Non-Cognitive Skills → Cognitive Performance path is also marginally stronger for girls (β = 0.007) than for boys (β = 0.006). The chain mediation involving ECEC → Non-Cognitive Skills → Truancy → Cognitive Performance shows a stronger effect for boys compared to girls (β = 0.005 for girls; β = 0.006 for boys). The slight difference in indirect effect between boys and girls may reflect gender variations in how non-cognitive skills and truancy influence cognitive performance. For instance, females show slightly stronger mediation through truancy, which could be due to gender-specific patterns in classroom engagement, social behaviors, or the development of perseverance and cooperation, which shape how ECEC participation indirectly affects learning.

## 5. Discussion

Reinforcing students’ cognitive performance has drawn increasing attention recently. Many scholars have investigated the possible factors that influence cognitive performance, and some of them suggested that equal access to ECEC serves as a tool for cognitive development. However, little is known about how non-cognitive skills and truancy mediate the link between ECEC and cognitive performance. The current study therefore leveraged the PISA 2022 student dataset, focusing on mathematics, reading, and science cognitive tests to analyze the intricate relationship among ECEC, non-cognitive skills, truancy, and cognitive performance, and revealed direct and significant relationships between ECEC and non-cognitive skills, ECEC and truancy, ECEC and cognitive skills, non-cognitive skills and truancy, non-cognitive skills and cognitive performance, and truancy and cognitive performance. The study also found that non-cognitive skills and truancy significantly mediate the relationship between ECEC and cognitive performance.

The study observed that cognitive performance is influenced positively by ECEC, which is an indication that promotion of ECEC programs is essential for enhancement in adolescents’ learning outcomes. This finding agrees with prior research and suggests that efforts to improve adolescents’ cognitive abilities should not be concentrated solely at the primary or secondary school levels (Betancur et al. 2024). The early learning experiences children have during ECEC are crucial for shaping their thinking skills, laying the groundwork for their future academic abilities. This relationship is slightly stronger for girls, suggesting that they may gain more benefits from early educational exposure. Research shows that girls often respond better to organized learning settings and social-emotional experiences at younger ages, which might enhance the cognitive benefits they get from ECEC (Saitadze 2021; Ward et al. 2022). By nurturing essential skills such as language development, early numeracy, attention regulation, and critical thinking (Liu et al. 2025a), ECEC programs can equip children with the mental tools necessary to navigate more complex learning environments in later years.

The direct correlation between ECEC and non-cognitive skills aligns with the literature, which suggests that students who received early childhood education and care expanded on their non-cognitive skills (Korsvold and Nygård 2022; Thümmler et al. 2022). Possibly, ECEC programs shape students’ skills and emotional reinforcement as they engage in active and positive interactions with their teachers, caregivers, and peers. For instance, the play-based nature of ECEC programs encourages cooperation, perseverance, and curiosity, which comes as students are inclined to winning and teachers are determined to ensure harmony (Saitadze 2021). The consistent engagement in this skills development routine likely forms part of the student’s behavior as he or she develops into an adolescent. Moreover, the stronger association observed among female students may reflect gender variances in receptiveness to early learning environments. Research suggests that girls often exhibit higher levels of compliance and social sensitivity at younger ages, which may make them more receptive to the socio-emotional components embedded in ECEC programs (Alam et al. 2024). This heightened responsiveness could explain why early educational interventions seem to yield more robust gains in non-cognitive development for female students compared to their male peers.

Similarly, the finding between ECEC and truancy echoes previous studies that explained that ECEC reduces certain negative behaviors that may be observed among students in adolescence (Schmidt and Li 2025; Juaristi et al. 2024; Riggleman et al. 2025). Students may engage in truancy due to a lack of interest or motivation for learning. However, participation in ECEC programs changes their perception about schooling and reduces the chances of truancy. In addition, ECEC nurtures in young learners a sense of belonging and structured time management, which probably translates into optimistic attitudes (Wei et al. 2023). The behavioral regulation learned in early childhood may act as a buffer against future risk behaviors, including skipping school or becoming disengaged. In this study, the stronger impact of ECEC on reducing truancy among female students suggests that girls may be particularly sensitive to the relational and affective components of early schooling, which could enhance their long-term school commitment. These finding emphasizes the preventive potential of ECEC programs in addressing attendance-related issues and highlight the importance of expanding access to early education, particularly in communities where truancy is prevalent (Zhou et al. 2023).

Findings illustrate that non-cognitive skills development is key for reducing truancy among students. One possible explanation is that possession of high-level perseverance, curiosity, assertiveness, stress management, and emotional control could help students overcome any obstacle or difficulty that may stir up truancy in them (Evans et al. 2023; Sangsuk and Thipchart 2023; Williams et al. 2022). Students may be motivated intrinsically to be resilient and committed to academic routines despite challenges. This influence would also reduce the adverse effect that truancy may have on cognitive performance. Truancy in itself is a threat to overall academic achievement, as it reduces the number of contact hours learners have with their teachers and to curriculum content (Boccio et al. 2025). The chain pathway underscores the mediating role of school attendance in translating non-cognitive development into academic success, suggesting that fostering non-cognitive competencies early on can have a dual benefit: reducing risky behavioral outcomes like truancy while supporting stronger cognitive performance in adolescence. Although the model benefits girls to a larger extent, the sequential pathway was especially pronounced among boys, highlighting the gender-sensitive nature of the ECEC experience and the behavioral benefits it offers boys in particular. These findings not only support existing theories on the long-term benefits of socio-emotional development but also reinforce the importance of integrated policy efforts that promote early education, build non-cognitive skills, and ensure regular school attendance as a coordinated strategy for boosting adolescent learning outcomes (Korsvold and Nygård 2022; Thümmler et al. 2022).

## 6. Implications

Overall, findings reinforce the call for greater investment in accessible ECEC programs, particularly in contexts where academic underperformance and gender disparities persist (Liu 2024). Recognizing and leveraging the differential pathways through which boys and girls benefit from ECEC can help inform more equitable and targeted interventions to improve long-term cognitive outcomes for all learners. The results also underscore the critical role of ECEC not only in academic preparation but also in laying the foundation for long-term behavioral and emotional competencies. For example, ECEC curricula can be designed to explicitly develop non-cognitive skills such as perseverance, cooperation, curiosity, and emotional regulation, while teacher training programs can include practical strategies to engage students, reduce truancy, and support positive classroom behaviors. Consequently, integrating explicit socio-emotional learning components into ECEC curricula and ensuring equitable access to ECEC programs may significantly enhance students’ non-cognitive skillsets, particularly in under-resourced communities. Gender-sensitive approaches within early childhood education, such as fostering inclusive pedagogies and relational learning, could further enhance its effectiveness for all students. Finally, the findings reinforce the importance of policy interventions that prioritize universal access, continuous teacher training, and age-appropriate curricular frameworks that stimulate cognitive engagement from an early age. Strengthening the ECEC sector through these targeted strategies could yield long-term academic dividends and play a pivotal role in raising the overall academic achievement of school-age children.

## 7. Limitations and Suggestions for Future Research

The current study has several limitations that should be considered when interpreting the findings. First, the study leveraged a cross-sectional dataset, which limits the ability to make causal inferences about the observed relationships. Future research should consider longitudinal designs to fully capture the developmental sequences implied in the proposed chain model. Second, the self-reported nature of the data may introduce social desirability or recall biases, potentially affecting accuracy. Mixed-methods approaches in future studies could provide a more comprehensive investigation of these relationships. Third, although PISA provides a large, internationally comparable dataset, the generalizability of findings may vary across cultural and policy contexts, particularly regarding access to ECEC programs. Alternative explanations may also account for some findings; for example, cultural and systemic differences across the 65 countries might influence both ECEC access and student outcomes. Similarly, unmeasured family characteristics, such as parental involvement, socio-economic resources, or home learning environments, could affect both early childhood education participation and later cognitive and non-cognitive outcomes. Finally, school-level factors beyond ECEC availability, including teacher quality and overall school climate, may also contribute to the effects observed. Future studies should consider these contextual factors to strengthen understanding of the relationships between ECEC, non-cognitive skills, truancy, and cognitive performance.

## 8. Conclusions

The current study explored the intricate relationships among ECEC, non-cognitive skills, truancy, and cognitive performance and found that ECEC improves non-cognitive skills, reduces truancy, and enhances cognitive performance in later stages of education. This study also established a positive and significant relationship between non-cognitive skills and cognitive performance. Inverse relationships were observed between ECEC and truancy, non-cognitive skills and truancy, and truancy and cognitive performance. Notably, the mediation analysis revealed that non-cognitive skills and truancy significantly mediate the relationship between ECEC and cognitive performance. The study concludes that ECEC is critical for ensuring improvement in cognitive performance and academic success in general. Hence, policymakers and stakeholders should implement key policies that would ensure access to ECEC programs, especially for children in disadvantaged communities.

## Figures and Tables

**Table 1:** Demographic characteristics of students.

Variables	Mean	Std. Dev
Female	0.49	-
Age	15.79	0.29
Grade Level	12.33	14.99
Native-born	0.81	-
Mother’s level of education	0.56	-
Index of economic, social, and cultural status (ESCS)	3.81	19.83

**Table 2:** Latent measurement characteristics of non-cognitive skills.

Item	Mean	SD	Factor Loadings
Perseverance	0.006	0.993	0.691
Stress resistance	−0.0251	0.984	0.845
Emotional control	−0.0043	0.972	0.862
Assertiveness	−0.0163	0.994	0.585
Curiosity	0.055	1.026	0.784
Cooperation	0.023	1.017	0.556

Note: Non-cognitive skills’ Cronbach’s ∝ is 0.703, KMO is 0.824, Bartlett’s test-of-sphericity 180,000 (df = 15, *p* = 0.001).

**Table 3:** Latent measurement characteristics of truancy.

Items	Mean	Std. Dev	Factor Loadings
ST062Q01TA ‘I <skipped> a whole school day.’	1.31	0.901	0.763
ST062Q02TA ‘I <skipped> some classes.’	1.33	0.953	0.782
ST062Q03TA ‘<I arrived late> ‘at school’	1.69	1.051	0.914

Note: Truancy’s Cronbach’s alpha is 0.841, KMO is 0.823, Bartlett’s test-of-sphericity (df = 3, *p* = 0.001).

**Table 4:** Measurement characteristics of cognitive performance.

Domains	Mean	Std. Dev	Factor Loadings
Reading	438.225	109.508	0.920
Mathematics	440.875	101.841	0.948
Science	450.463	105.244	0.962

Note: Cognitive Performance’s Cronbach’s alpha is 0.965, KMO is 0.869, Bartlett’s test-of-sphericity (df = 3, *p* = 0.001).

**Table 5:** Structural equation model results.

Pathways	Std. β	Z-Value	*p*-Value	[95% Conf. Interval]
Direct effects				
ECEC → Truancy	−0.054	−22.11	0.001	[−0.059, −0.049]
ECEC → NONCOG	0.040	15.36	0.001	[0.035, 0.045]
ECEC → COG	0.057	28.20	0.001	[0.053, 0.061]
NONCOG → Truancy	−0.177	−56.68	0.001	[−0.183, −0.170]
Truancy → COG	−0.223	−90.88	0.001	[−0.228, −0.218]
NONCOG → COG	0.160	59.48	0.001	[0.154, 0.165]
Indirect effects				
ECEC → Truancy → COG	0.012	21.397	0.001	[0.011, 0.013]
ECEC → NONCOG → COG	0.006	15.539	0.001	[0.006, 0.007]
ECEC → NONCOG → Truancy → COG	0.007	37.10	0.001	[0.007, 0.008]

**Table 6:** Structural equation model results based on gender.

Pathways	Std. β	Z-Value	*p*-Value	[95% Conf. Interval]
Direct effects				
ECEC → Truancy	−0.062/−0.048	−17.86/−13.95	0.001/0.001	[−0.069, −0.055]/[−0.055, −0.041]
ECEC → NONCOG	0.045/0.036	12.16/9.73	0.001/0.001	[0.038, 0.052]/[0.029, 0.043]
ECEC → COG	0.058/0.054	20.32/18.70	0.001/0.001	[0.052, 0.063]/[0.048, 0.059]
NONCOG → Truancy	−0.201/−0.149	−45.12/−34.72	0.001/0.001	[−0.209, −0.192]/[−0.158, −0.140]
Truancy → COG	−0.222/−0.223	−62.51/−65.26	0.001/0.001	[−0.229, −0.215]/[−0.229, −0.216]
NONCOG → COG	0.145/0.172	37.83/46.59	0.001/0.001	[0.138, 0.153]/[0.166, 0.180]
Indirect effects				
ECEC → Truancy → COG	0.014/0.011	17.09/13.60	0.001/0.001	[0.012, 0.015]/[0.009, 0.012]
ECEC → NONCOG → COG	0.007/0.006	11.553/9.512	0.001/0.001	[0.005, 0.008]/[0.005, 0.007]
ECEC → NONCOG → Truancy → COG	0.005/0.006	27.47/24.26	0.001/0.001	[0.005, 0.006]/[0.006, 0.007]

Note: All results are presented by Female/Male.

## Data Availability

Data used in this study can be publicly accessed from the OECD data repository (https://www.oecd.org/en/data/datasets/pisa-2022-database.html, accessed on 12 May 2025), with permission of the Organization for Economic Cooperation and Development (OECD).
